# To help others or not: A moderated mediation model of emotional dissonance

**DOI:** 10.3389/fnhum.2022.893623

**Published:** 2022-08-04

**Authors:** Ling Hu, Stanley Y. B. Huang, Hung-Xin Li, Shih-Chin Lee

**Affiliations:** ^1^Department of Finance, Hsing Wu University, New Taipei City, Taiwan; ^2^Ming Chuan University, Master Program of Financial Technology, Taipei City, Taiwan; ^3^Department of Logistics Management, National Defense University, Taipei City, Taiwan; ^4^Department of Finance, Chihlee University of Technology, New Taipei City, Taiwan

**Keywords:** emotional dissonance, emotional leadership, help other behavior, work-to-family conflict, emotion regulation

## Abstract

This article proposes a moderated mediation model of emotional dissonance. In the model, emotional leadership negatively affects emotional dissonance, which, in turn, negatively affects helping behavior. Furthermore, the negative effect of emotional dissonance is assumed to be moderated by work-family conflict. Direct effects from both emotional leadership and work-family conflict to helping other behavior are also considered. Previous studies have neglected the mechanism of emotional dissonance, but this paper fills the gap with a moderated mediation model of emotional dissonance. This article not only provides an incremental contribution to the emotional dissonance literature but also suggests means by which companies might enhance employe helping behaviors in order to achieve greater organizational efficiency.

## Introduction

The service industry has become the main industrial structure in countries around the world (The World Bank, 2016; Indregard et al., 2018), so exploring emotion regulation mechanisms has become a major topic in the industry (Geisler et al., 2019; Madrid et al., 2020). However, due to the current highly competitive service industry environment, employes often experience emotional dissonance because their emotional resources are insufficient to meet the needs of emotional work (Park et al., 2019). Emotional dissonance denotes the degree to which individuals show work emotions that are inconsistent with their values (Zapf and Holz, 2006). For example, organizations expect employes to smile and be professional to customers, but such behaviors might not necessarily be in line with the employes’ own values, resulting in emotional dissonance. That is, some employes may have the value that, as long as the customer’s problems are dealt with, there is no need to show additional emotional behaviors, such as showing a smile or professionalism.

To fill this gap, this article employs emotional leadership (Grandey, 2000) as an antecedent to emotional dissonance. Indeed, the emotional leadership of supervisors can shape an employe’s values to conform more to a company’s expectations (Huang et al., 2021). In this way, the company can deliver expectations of work emotions to the employe through emotional leaders, thus decreasing the inconsistency between individuals’ values and work emotions (i.e., emotional dissonance). For example, by smiling and acting professionally, emotional leaders can convey the behaviors that the organization wants employes to show. Hence, emotional leaders can shape employes’ values, which ultimately lead to employes’ willingness to smile and be professional themselves. Emotional leadership denotes the degree to which a leader employs consideration, understanding, and respect to transform employes’ emotions to meet organizational expectations (Grandey, 2000).

In addition, emotional dissonance can lead to low levels of helping other behaviors in employes because these employes rarely perform helping other behaviors that drain personal resources. Previous research has also examined work-family conflict and its impact on employe negative behavior (Li et al., 2021; Zhang et al., 2021) because of the highly competitive service industry environment (Obrenovic et al., 2020). However, the moderating role of work-family conflict has not been examined in the service industry environment because work-family conflict is almost regarded as a driving factor of negative employe behavior. This article argues that work-family conflict can worsen the relationship between emotional dissonance and helping other behaviors, because employes with high levels of work-family conflict should lead to more resource scarcity problems, thereby further exacerbating the effect of emotional dissonance on helping other behaviors. For example, when employes have emotional dissonance, their values are inconsistent with the emotional performance expected by the organization. However, to continue to work, employes must display emotional behaviors that do not conform to their values but meet the organization?s expectations, such as smiling and professionalism. Because employes may suppress their anger or sadness to show these emotional behaviors, this will inevitably consume more emotional resources. Moreover, if these employes have stronger work-family conflict, they have over lower resources to show helping other behaviors.

## Literature review

**FIGURE 1:**
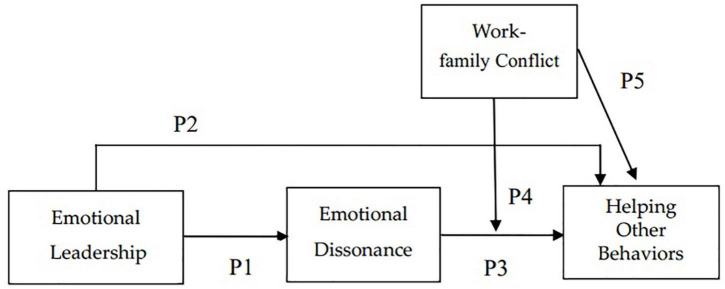
A moderated mediation model of emotional dissonance.

This paper proposes a new framework in Figure 1 that emotional leadership influences emotional dissonance, which then influences helping other behaviors, in the manner that is moderated by work-family conflict.

(1). Emotional leadership and emotional dissonance

Because an emotional leader can transform the original values of employes into the expected ones that meet the need of an organization by caring, understanding, and respecting the needs of employes (Huang et al., 2021), an emotional leader can pass the value expected by the organization to the employes (Shamir et al., 1993), which will eventually lead to the employes’ values, meeting organizational expectations of work emotions. That is to say, emotional dissonance occurs when employes display work emotions that are inconsistent with their values (Zapf and Holz, 2006), and emotional leadership can reduce this dissonance through an emotional leadership process. Indeed, a leader is expected to manage the employes in a meaningful way that meets the expectations of the organization based on the theory of meaning management (Smircich and Morgan, 1982), so these employes should show emotional behaviors that are expected by organizations. Therefore, he/she will transmit the expected value of the organization to the subordinates, and also shape the work behavior of the subordinates to meet the expectations of the organization, hence reducing the emotional dissonance of these employes.

However, to date, there have been no surveys to explore that relationship. In addition, emotional leadership is a work resource that can support employes’ emotional needs (Totterdell and Holman, 2003), because the emotional leadership process can guide the optimal management of employes’ emotions, thereby generating more emotional resources for being used by employes. In the same vein, emotional leadership can shape the work values of employes into the expected organizations’ values, so it will increase employes’ positive behaviors more generally, such as helping others. Therefore, this article proposes the two propositions as follows:

Proposition 1: Emotional leadership can decrease emotional dissonance.
Proposition 2: Emotional leadership can increase helping-other behaviors.

(2). Emotional dissonance and helping other behaviors

Emotion regulation is an element of emotional work, which includes various positive and negative emotions of employes (Zapf and Holz, 2006). An important dimension of emotion regulation is emotional dissonance (Zapf, 2002), which refers to the fact that employes express emotions that are in line with a company’s expectations but are contrary to their emotional values. When employes are immersed in a state of emotional dissonance, they exhibit various negative behaviors, such as emotional exhaustion, absenteeism, and illness (Indregard et al., 2016). Indeed, when employes’ emotional resources are unable to cope with job demands, they may display emotional exhaustion or absenteeism to preserve the last few resources. Since these employes have few resources, they may conserve resources by reducing helping-other behaviors. Employes are thoughtful in allocating key resources (Macan, 1994), and they should reduce non-performance-related helping behaviors in the absence of personal resources.

Proposition 3: Emotional dissonance can decrease helping other behaviors.

(3).Work-family conflict, emotional dissonance, and helping other behaviors

Work-family conflict denotes a “form of inter-role conflict in which the role pressures from the work and family domains are mutually incompatible in some respect” (Greenhaus and Beutell, 1985, p. 777). That is to say, employes invest most of their resources in their work because these employes need to earn money to maintain their families. Based on the conservation of resources theory (Hobfoll, 1989), this will cause these employes to go home with very few resources. If employes occur high levels of work-family conflict, it follows that these employes do not have sufficient resources to meet work and family needs. Therefore, a high level of work-family conflict will further deteriorate the relationship between emotional dissonance and helping-other behaviors, because these employes with high-level emotional dissonance have little resources to yield helping-other behavior.

In the same vein, if employes do not have sufficient resources to meet work and family needs, these employes must reduce helping-others behavior to hold their resources. Therefore, this article proposes the two propositions as follows:

Proposition 4: Work-family conflict can moderate the relationship between emotional dissonance and helping-other behaviors
Proposition 5: Work-family conflict can decrease helping-other behaviors.

## Discussion

This article proposes a new model of emotion regulation to predict the cognitive processes underlying helping behaviors, which is unique and cannot be explained by past models. Emotion regulation has emerged as an important antecedent of employe performance, as many negative employe behaviors are associated with the demands incurred by emotion regulation, such as emotional exhaustion (Alsalhe et al., 2021), counterproductive behaviors (Huang et al., 2021), and disorders of emotion (Greening et al., 2014). However, past investigations have not explored the relationship between emotional leadership and emotional dissonance to predict helping behaviors. They have also not considered the role of work-family conflict as an important moderating variable, which could significantly advance the literature on emotion regulation.

In addition, contemporary businesses must develop strategies to deal with the emotional issues of employes, as employes are an important source of performance and competitive advantage. Indeed, emotional problems lead to many negative employe behaviors. This article considers emotional leadership as a significant organizational leadership mechanism because it should not only alleviate emotional dissonance but also increases helping behaviors. Therefore, emotional leadership could be regarded as important educational and training content for cultivating executive leadership.

Finally, although this article proposes an emotion regulation model, future investigations should adopt empirical data to verify the model’s validity. Moreover, a multi-country sample should be used to verify the external validity of the model.

## Author contributions

All authors listed have made a substantial, direct, and intellectual contribution to the work, and approved it for publication.
